# Supplementary figures and images for: Rift Valley Fever Outbreak Investigation Associated with a Dairy Farm Abortion Storm, Mbarara District, Western Uganda, 2023
Source: Viruses. 2025 Jul 19;17(7):1015. doi: 10.3390/v17071015 (PMC12298680; doi:10.3390/v17071015)

S segment

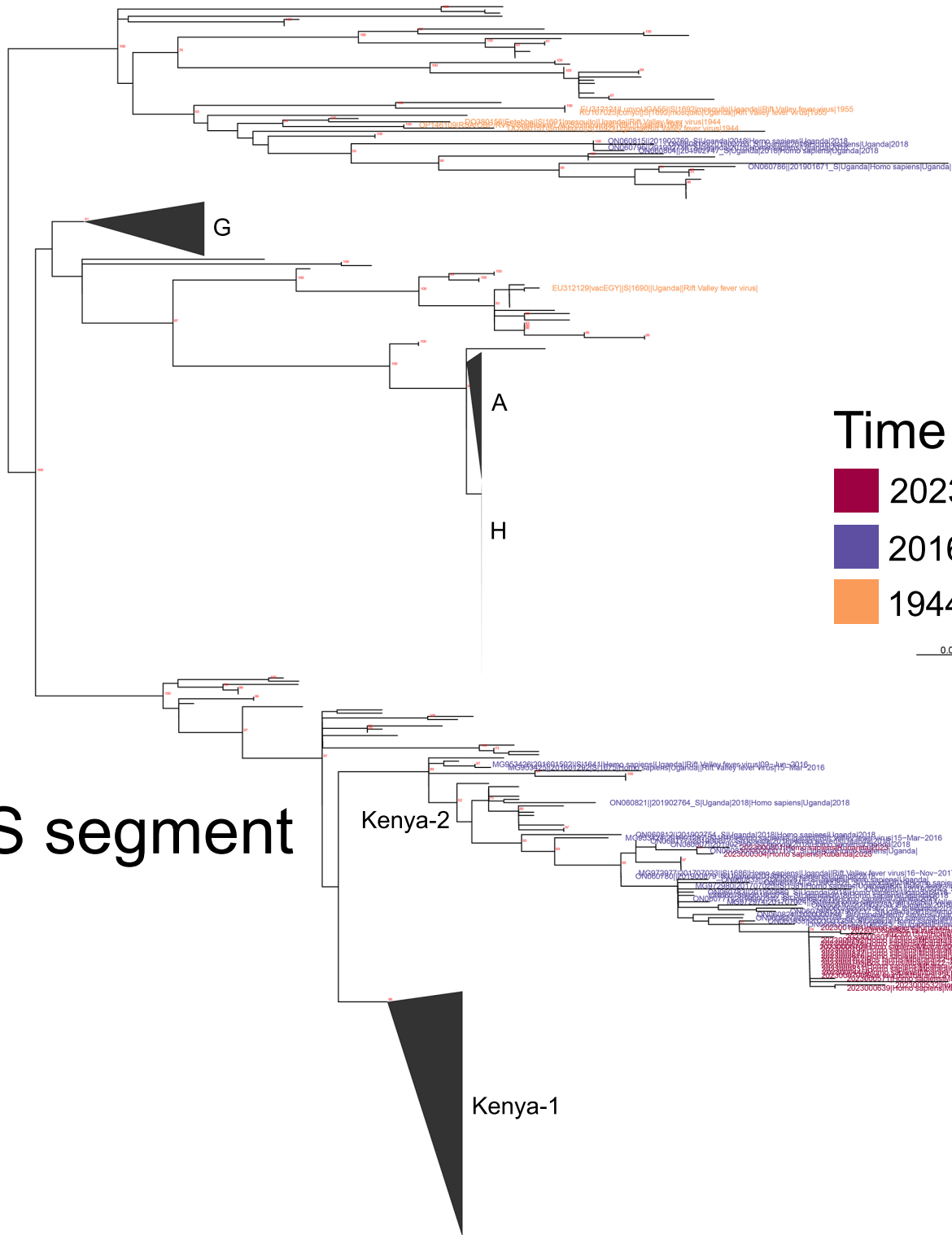

Time Frame

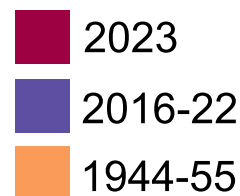

0.003

Supplement: Supplementary file 1 [file viruses-17-01015-s001.zip › Suppl_Figure_S2_S_segment.pdf]
