# Supplementary material for: Rift Valley Fever Outbreak Investigation Associated with a Dairy Farm Abortion Storm, Mbarara District, Western Uganda, 2023
Source: Viruses. 2025 Jul 19;17(7):1015. doi: 10.3390/v17071015 (PMC12298680; doi:10.3390/v17071015)
Supplement: Supplementary file 1 [file Suppl_Figure_S1_M_segment.pdf › Suppl_Figure_S1_M_segment.pdf]

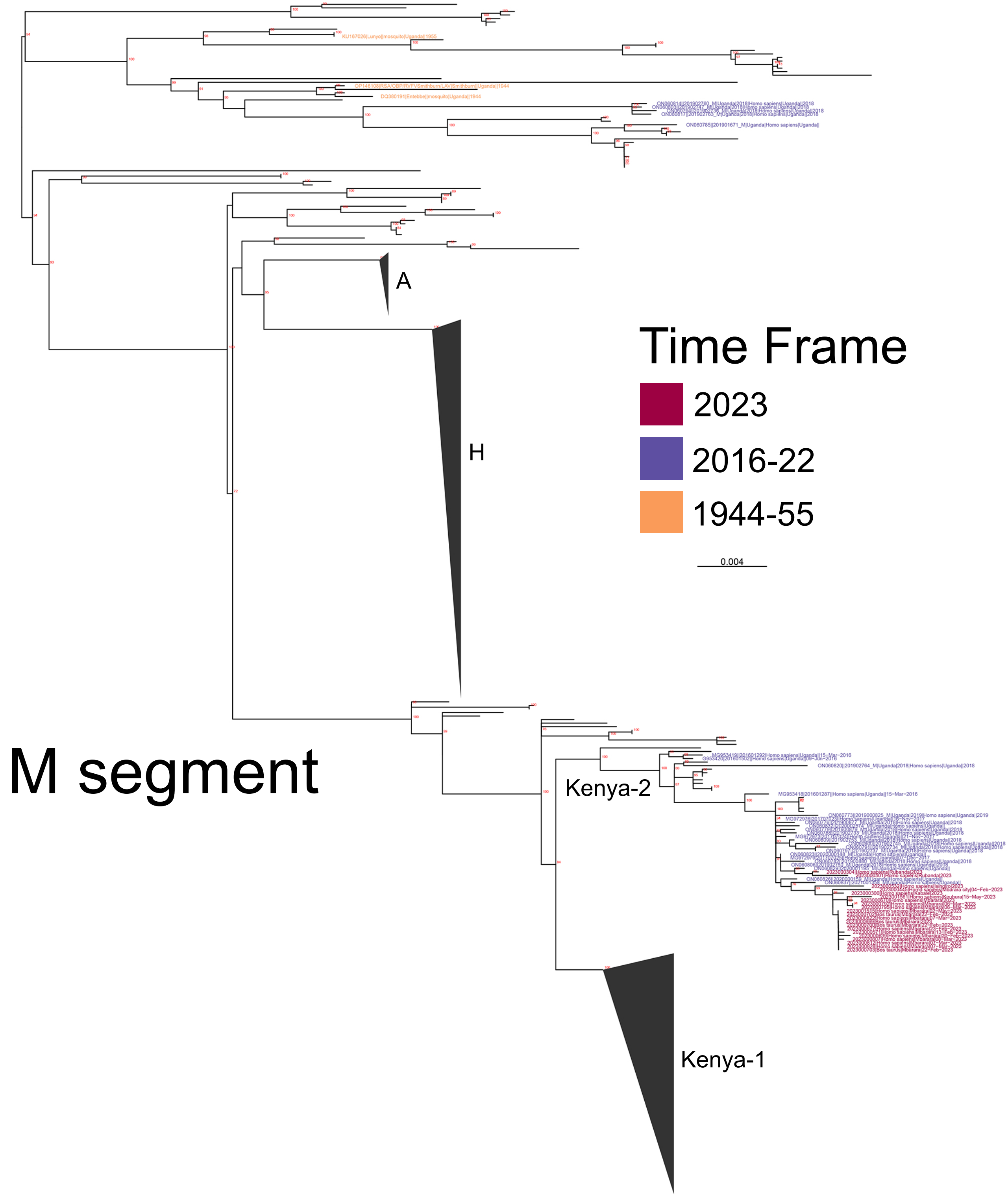

M segment
A
H
Kenya-2
Kenya-1
Time Frame
2023
2016-22
1944-55
0.004
